# Phase Transitions of Oppositely Charged Colloidal Particles Driven by Alternating Current Electric Field

**DOI:** 10.1021/acsnano.0c04095

**Published:** 2021-02-12

**Authors:** Bin Li, Yong-Lei Wang, Guang Shi, Yangyang Gao, Xinghua Shi, Clifford E. Woodward, Jan Forsman

**Affiliations:** † Laboratory of Theoretical and Computational Nanoscience, CAS Key Laboratory for Nanosystem and Hierarchy Fabrication, CAS Center for Excellence in Nanoscience, National Center for Nanoscience and Technology, Chinese Academy of Sciences, Beijing 100190, China; ‡ Theoretical Chemistry, Chemical Center, Lund University, P.O. Box 124, S-221 00 Lund, Sweden; § School of Chemical Engineering and Technology, Sun Yat-sen University, Zhuhai 519082, China; ⊥ Department of Materials and Environmental Chemistry, Arrhenius Laboratory, Stockholm University, SE-106 91 Stockholm, Sweden; ∥ Department of Chemistry, University of Texas at Austin, Austin, Texas 78712, United States; ¶ Key Laboratory of Beijing City on Preparation and Processing of Novel Polymer Materials, Beijing University of Chemical Technology, Beijing 10029, China; # State Key Laboratory of Organic−Inorganic Composites, Beijing University of Chemical Technology, Beijing 10029, China; △ School of Physical, Environmental and Mathematical Sciences, University College, ADFA, University of New South Wales, Canberra, ACT 2600, Australia

**Keywords:** colloidal particles, phase transition, alternating current electric field, overdamped Langevin simulation, non-equilibrium thermodynamics

## Abstract

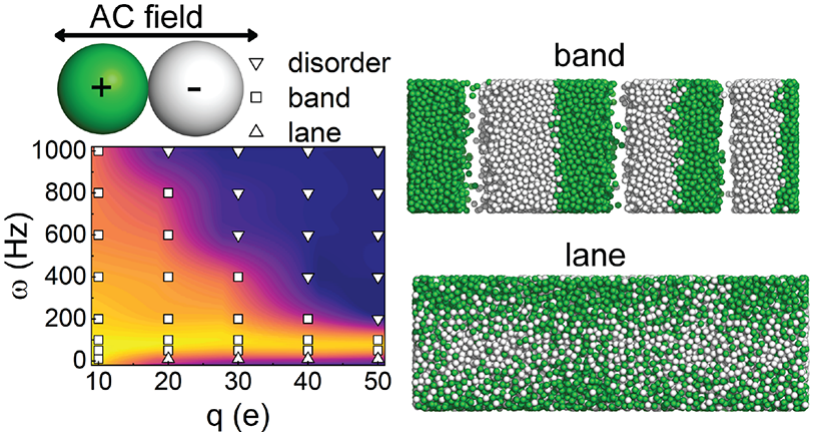

We study systems containing oppositely charged colloidal particles under applied alternating current electric fields (AC fields) using overdamped Langevin dynamics simulations in three dimensions. We obtain jammed bands perpendicular to the field direction under intermediate frequencies and lanes parallel with the field under low frequencies. These structures also depend upon the particle charges. The pathway for generating jammed bands follows a stepwise mechanism, and intermediate bands are observed during lane formation in some systems. We investigate the component of the pressure tensors in the direction parallel to the field and observe that the jammed to lane transition occurs at a critical value for this pressure. We also find that the stable steady states appear to satisfy the principle of maximum entropy production. Our results may help to improve the understand of the underlying mechanisms for these types of dynamic phase transitions and the subsequent cooperative assemblies of colloidal particles under such non-equilibrium conditions.

It is known that soft condensed matter (or *soft matter* for short) can assemble into an array of nontrivial structures under non-equilibrium conditions.^1−5^ These can be considered dynamic phase transitions, which may be readily observed over time scales that can be realized in experiments. Indeed, a variety of soft matter systems have been studied over a number of years, including effective self-propelling particles,^6,7^ polymer blends,^8,9^ and colloids, among others.^10−14^

Colloidal dispersions, in particular, occur in many areas of interest, *e.g.*, in biological and environment systems, to name a few,^15,16^ and respond sensitively to the application of external fields.^17−20^ It is quite common that colloidal dispersions are stabilized by surface charges, which are screened by smaller ions in the surrounding solution. These ions may specifically adsorb to the colloidal surfaces or else form diffuse electrical double layers, which act to diminish the magnitude of colloid–colloid interaction in the dispersion. Mixtures of oppositely charged colloidal particles form a soft matter analogue of electrolyte solutions, albeit the interactions follow a Yukawa rather than Coulombic form, due to the screening of the mobile ions. When the Debye length becomes very short, one can consider such systems as “colored” rather than “charged”, and the colloidal particles have short-ranged mutual interactions, even while external fields will force different colored (charged) colloids in opposite directions. Such systems, in the presence of a constant driving field, have been well-studied theoretically.^21−23^ It has been found that a lattice-gas model for the two-colored particle system has a propensity to form so-called “jammed” structures, wherein interfaces between layers of similarly colored particles form perpendicular to the driving field.^22^

Löwen and co-workers have simulated mixtures of charged colloidal particles driven by external fields in a continuum two-dimensional (2D) space. They also obtained ordered steady-state structures for such systems.^24−26^ When the field is oscillatory, it appears that both jammed and so-called “laned” structures may form, depending upon factors such as the magnitude of the driving field and its oscillatory frequency. For charged particles, the magnitude of the surface charge also plays a role. Laning generally occurs for a high enough field strength and a low frequency of oscillation.^24^ Here, the colloidal mixture forms long rows (lanes) of similarly charged particles moving parallel to the field. Some evidence points to this being a second-order transition from the uniform randomly mixed state, with the correlation length of the lanes growing exponentially with the field strength.^27^ However, more recent studies suggest that this observation may be an artifact of the slow dynamics of lane formation from an initially random configuration. A correspondence between density-dependent dynamics and attractive fluids at equilibrium suggests that laning is instead a first-order (discontinuous) transition.^28^ Related theoretical studies have considered driven colloidal particles in channels and in spatially periodic fields.^29−31^

Experimental studies of the behavior of colloidal particles in external fields have also been quite prevalent.^32^ Of particular relevance to the work presented here are experiments by Vissers *et al.* on charged colloids, where both jammed^33^ and laned^34^ steady states were observed in the presence of an applied AC field in three-dimensional (3D) systems. On the other hand, comparisons with such experimental work have usually involved simulations performed with 2D planar systems. In this work, we revisit the problem of charged colloidal particles in the presence of an electrolyte and an AC electric field. However, unlike most previous work, we will carry out a simulation study of the dynamic behavior of this system in 3D.

It is well-known that for equilibrium systems the phase behavior can be crucially dependent upon the dimensionality of the allowed fluctuations. For example, fluctuations of interfaces in 1D systems preclude phase separation. Furthermore, (infinite) 2D interfaces can also be subjected to unconfined capillary waves, leading to divergences in the interfacial width. Here, we investigate the propensity of the driven colloidal system in 3D to form steady-state jammed and laned structures seen in 2D studies and to determine the putative phase boundaries as a function of particle charge, as well as the strength and frequency of the driving fields.

## Results and Discussion

### Phase Diagrams

We modeled binary mixtures of oppositely charged colloidal particles with a range of charge magnitudes, *q*, varying from ±10*e* to ±50*e*. The interactions between the colloids are assumed to have a screened-Coulomb (Yukawa) form. An AC electric field, **E**(*t*) = *E*_0_ sin(2πω*t*), is applied to the dispersion with frequencies ω ranging from 10 Hz to 1 kHz. The colloidal particles are assumed to be dispersed in a highly viscous solvent, which is mimicked using overdamped Langevin dynamics; see Supporting Information (SI). Hydrodynamic and electrofriction effects are ignored in this study. Details of the simulation setup are given in Models and Methods and in the SI.

**Figure 1:**
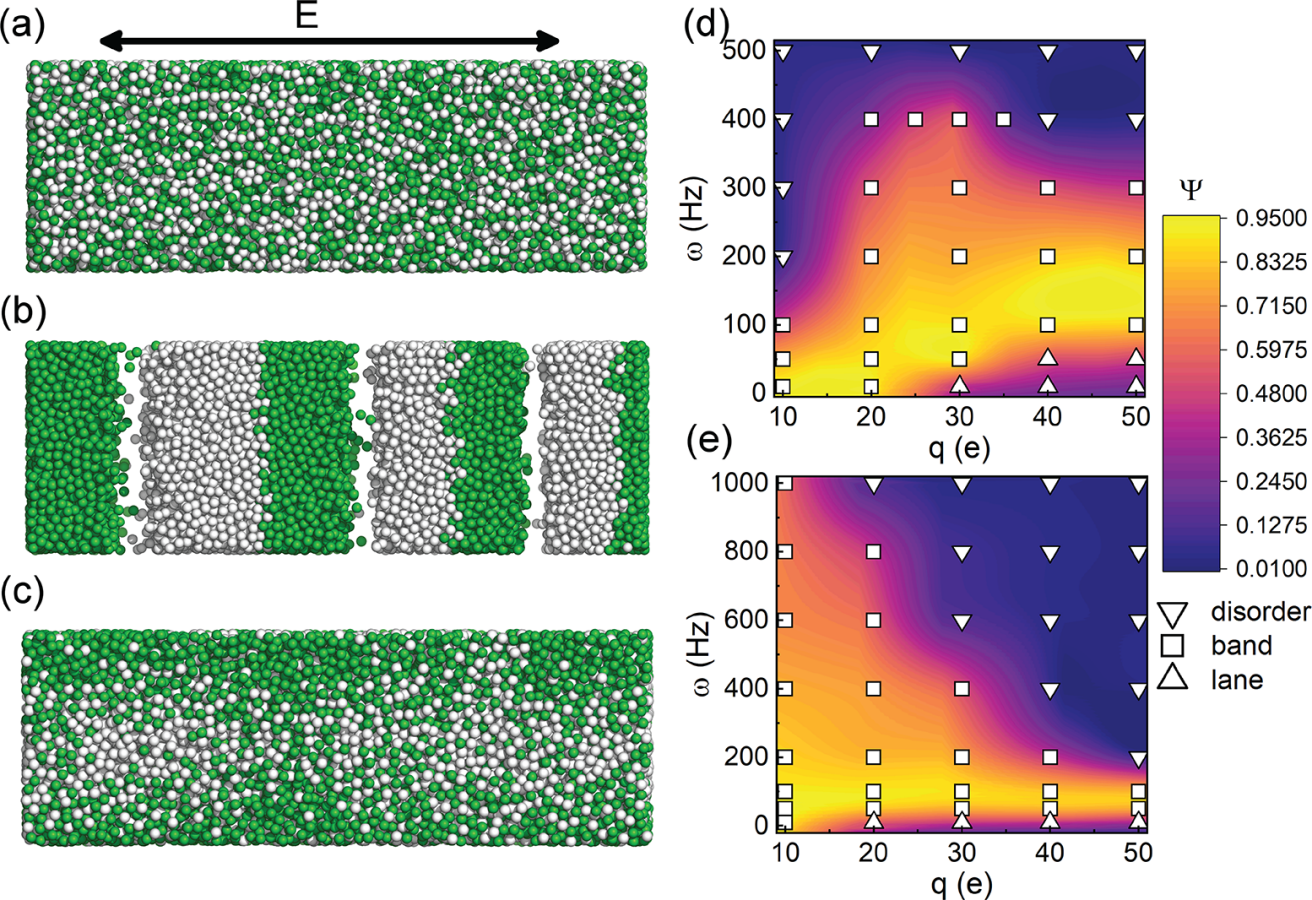
Representative configurational snapshots of colloidal particles with ±40*e* under applied AC fields, with *E*_0_ = 0.115 V/μm and different ω values: (a) 500 Hz, (b) 200 Hz, (c) 10 Hz. Positive particles are displayed as green, whereas negative particles are white. All snapshots in this report use the same color codes. The field direction is indicated by a double-headed arrow. Graphs (d) and (e) show approximate phase diagrams, including contour plots of the order parameter Ψ. The “jammed” steady-states are denoted as “band” structures in the labels. Graph (d) is obtained at a constant maximum field strength *E*_0_ = 0.115 V/μm, whereas the parameters in graph (e) are constrained by a constant maximum external force *F*_0_ = *E*_0_*q* = 0.552 pN.

Figure 1 shows representative snapshots of particles with *q* = ±40*e* in the presence of AC fields with the same maximum field strength, *E*_0_ = 0.115 V/μm, but with different ω values. For the largest frequency (500 Hz), only homogeneous steady-state structures could be obtained in the system (Figure 1a), but if we decrease ω to 200 Hz, the dispersion displays a jammed phase, with like-charged particles forming bands perpendicular to the applied field (Figure 1b). On the other hand, for even lower ω = 10 Hz, a laned steady-state structure is obtained instead. Here, the like-charged particles form columns parallel to the direction of the driving field^24^ (Figure 1c). Snapshots of the laned steady state from other angles are shown in Figure S1 in SI.

In order to quantify the structure of the system, we calculated an order parameter, Ψ, as follows. The quantity ψ_*i*_, for particle *i*, is obtained as^35,36^
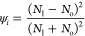
1where *N*_l_ or *N*_o_ is the number of neighboring particles of “like” or “opposite” charge (to particle *i*), respectively. The ψ_*i*_ values are then averaged over all particles to obtain Ψ. For the completely disordered (homogeneous) state, we have Ψ = 0, whereas for structures with regions where particles have the same charge, 0 < Ψ < 1. Larger Ψ corresponds to more ordered structure.

The putative dynamic phase diagram, describing the stable steady states, will ostensibly depend upon several variables, including field strength and frequency as well as particle charge and density. Here, we consider two “slices” through this multidimensional phase diagram. First (at fixed volume fraction of particles, see Models and Methods), we keep the absolute maximum field strength fixed at *E*_0_ = 0.115 V/μm and vary the values of *q* and ω. The stable phase regions were established by noting the steady states which evolved in the simulations over a transient period, starting from the uniform mixture. After this transient period, Ψ values were calculated at the end of each oscillating period and averaged over a simulated time of *t* = 2 s. During this time, the observed structure and total energy do not vary significantly over different periods of oscillation, which indicates the systems reach a steady state.

Figure 1d shows the resulting phase diagram, with varying ω *versusq*. Also shown is the corresponding contour plot of the order parameter Ψ. At a frequency of ω = 500 Hz, a homogeneous phase is obtained for all of the particle charges investigated. Jammed structures, with bands perpendicular to the field, are formed at ω = 400 and 300 Hz, where we observe a re-entrant behavior. If the absolute charge of the particles is lower than about 20*e*, the force is insufficient over an oscillation period of τ_0_ to bring about a dynamical instability, and the system remains homogeneous. Also, at an absolute charge above ∼35*e*, charge–charge repulsions between particles (which scales as *q*^2^) are able to inhibit the formation of ordered domains. For intermediate charges, the field is able to create bands of like-charged colloids corresponding to jammed steady-state structures. This re-entrant behavior is clearly reflected in the Ψ values, which show a nonmonotonic behavior with respect to the particle charge. At lower field frequencies (between ω = 100 and 200 Hz), jammed bands with greater Ψ values are obtained, with these values increasing with particle charge. This is due to a stronger coupling with the field, promoting phase separation. However, if instead ω < 100 Hz, laned structures (parallel to the field) are found in systems with a high particle charge. At these lower frequencies, the parallel interface of the jammed structures is disrupted by the strong field coupling, and instead, lanes (which extend over the length of the box) form over the longer field period.

As the external forces on colloidal particles depend upon their charge at a fixed field strength, the phase diagram Figure 1d implicitly includes a varying maximum external force *F*_0_ = *E*_0_*q*. Thus, we also considered a “constant force” slice through the full phase diagram, where we vary both ω and *q* as before, but the absolute maximum force is kept constant, *i.e.*, *F*_0_ = 0.552 pN (*E*_0_ and *q* vary inversely). The corresponding results are given in Figure 1e. This slice through the phase diagram illustrates the role played by the Coulomb repulsion between particles. That is, as we increase *q* for the same ω and force, the system tends to become disordered, due to the repulsion within regions of similar charge. This effect dominates earlier at high field frequencies, due to the shorter period over which the force is able to drive the separation of particles. That is, for larger charges the homogeneous phase has a greater cohesive energy, which means the driving field needs to be applied for a longer period in order to drive the phase separation. Thus, at low frequencies (ω = 100 and 50 Hz), these jammed bands are still observed, even at an absolute charge of 50*e*. On the other hand, at very high frequencies, ω = 1 kHz, the charged particles only oscillate locally within a relatively small region and the jammed band phase is not exhibited, except at the lowest charge. It is noteworthy that, for *q* = ±10*e*, the jammed band phase is seen under all frequencies investigated. However, the order parameters behave nonmonotonically. For example, the order parameter for the *q* = ±10*e* system at ω = 10 Hz is lower than that at ω = 50 Hz, perhaps because of some incipient laning.

Laned structures parallel with AC field direction are observed in the more highly charged system for a field frequency of ω = 10 Hz. We also investigated the system in a constant electric field (DC field) along the *z* direction, with a field strength *E*_c_ = *E*_0_/√2. In this case, only lane structures (parallel to field) are obtained. Representative snapshots are shown in Figure S2.

### Phase Transition Pathways

**Figure 2:**
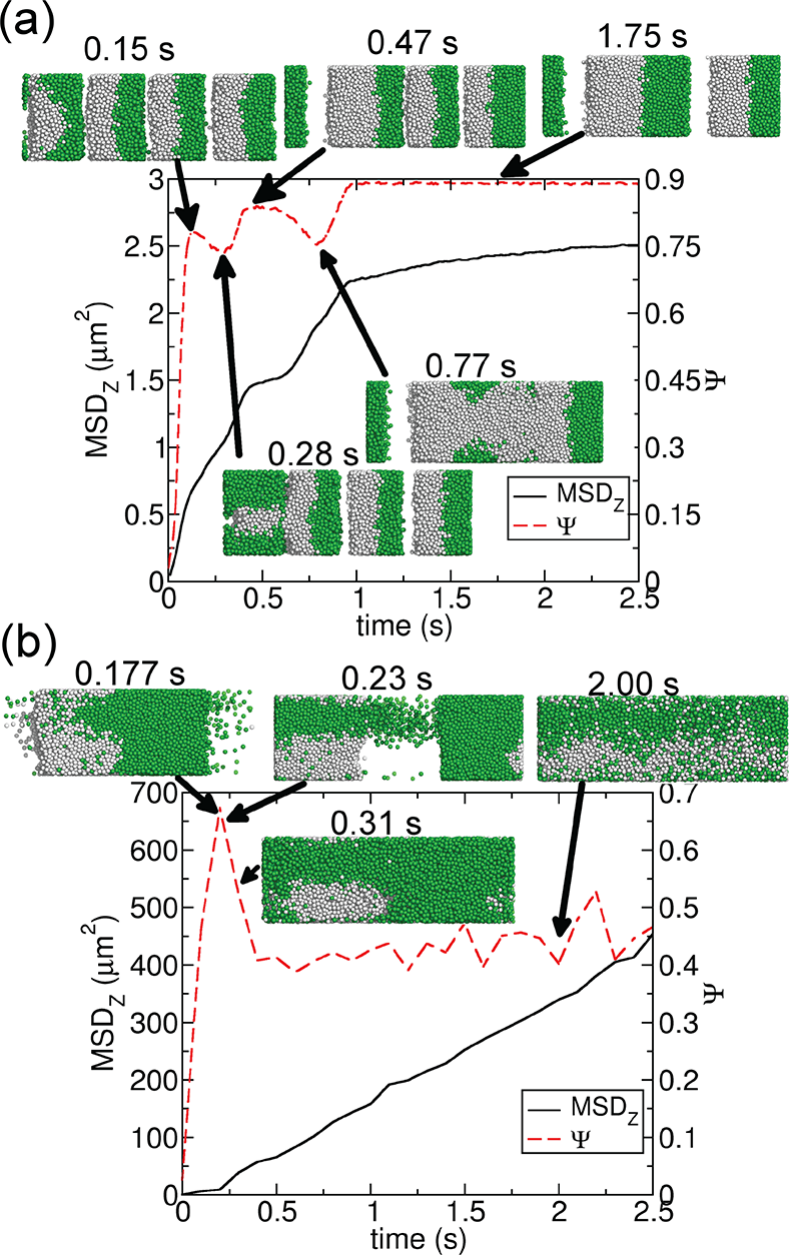
Time evolutions of MSD along the AC field direction and Ψ for systems containing particles with ±20*e*, at an AC field strength of *E*_0_ = 0.1725 V/μm. The snapshots are taken at the simulation times that are indicated by arrows: (a) ω = 100 Hz, (b) ω = 10 Hz.

In order to characterize the kinetic mechanisms by which the observed steady states evolved, we considered the time evolution of Ψ, as well as the mean squared displacement (MSD) along the field direction. This gave us some indication of the correlations between the structural evolution and dynamical properties of the colloidal particles. The MSD is determined by the equation^37^
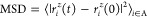
2where *r*_*i*_^*z*^(*t*) is the coordinate along the field direction of particle *i* of species A at time *t*. The results are given in Figure 2, together with the evolution of the order parameter Ψ, along with some representative snapshots from the simulations.

Figure 2a displays the time evolution of ±20*e* charged particles at a field strength *E*_0_ = 0.1725 V/μm and a frequency ω = 100 Hz. The system was started in the homogeneous mixed phase. Both the MSD and the Ψ initially increase rapidly, suggesting that the particles initially diffuse quickly to form like-charged clusters. The order parameter Ψ initially peaks at *t* = 0.15 s, and the corresponding snapshot shows that a jammed structure with relatively thin bands is initially formed. This is a relatively unstable jammed state, and the MSD curve is lowered only marginally after it is formed. In fact, diffusion in the period between 0.15 and 0.28 s remains somewhat high, and during this period, Ψ decreases until a local minimum occurs at 0.28 s. This can be attributed to the penetration of one band into another, momentarily creating a larger total interface between unlike charges and reducing Ψ. Between 0.28 and 0.47 s, regions of opposite charge move through each other and then merge to create thicker jammed bands, increasing Ψ (up to a new local maximum). This is another (metastable) jammed state corresponding to a transient period whereby the MSD (immediately after 0.47 s) is rather flat. However, this is followed quickly by an increase in the MSD as two bands (in the middle of the system) repeat the process of merging and reordering. The Ψ values oscillate again during this period. The Ψ value reaches a plateau after 1 s and does not increase any further. The MSD curve increases more slowly after 1 s, again because of the jamming effect. The penetration and merging appear to cease, as the period for the oscillation is insufficiently long to bring this about. Thus, jammed structures appear to emerge from the homogeneous phase *via* a series of metastable states with progressively thicker bands. The order parameter Ψ will oscillate as the system evolves, progressing to sequentially higher values until it reaches a steady state.

Figure 2b shows the pathway to a laned structure (from the homogeneous phase) for particles with charges ±20*e*, in a field with same maximum strength as above (*E*_0_ = 0.1725 V/μm) but with a frequency of ω = 10 Hz. Although the final phase is a laned structure, oriented parallel with the AC field direction, an intermediate jammed structure (perpendicular to the field) is observed to form initially corresponding to a maximum Ψ value at 0.177 s. This is also characterized by the initially flatter region in the MSD curve. This jammed structure starts to break up at around 0.23 s. This is initiated by a tilting of the interface leading to oppositely charged bands moving parallel with the field and temporarily opening of a void, which is subsequently filled at a later time. This process appears to form clusters of like charged particles which eventually percolate to form a laned steady state (after approximately 0.5 s). The MSD shows that the colloidal particles diffuse relatively quickly once the jammed structure is dismantled. Movies of the full trajectories for these two systems (Figure 2a,b) are available in SI. We do not observe an intermediate jammed structure perpendicular to field direction for highly charged particles at lower frequencies. The time evolutions of Ψ for particles with ±40*e* and ±50*e* at 10 Hz are shown in Figure S3.

**Figure 3:**
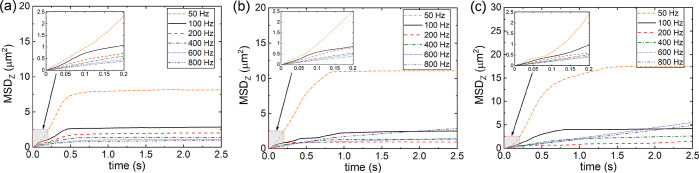
MSDs along the AC field direction for charged colloidal particles under different frequencies. (a–c) Results of ±10*e*, ±20*e*, and ±30*e* charged colloidal particles, respectively; the insets show the results of the first 0.2 s.

The MSDs for other systems are shown in Figure 3, with different charges and field frequencies (within the range of jammed and disordered steady states shown in Figure 1e). The diffusion is faster in the beginning at lower ω (insets of Figure 3). This tendency is the same for all of the investigated charges. On the other hand, the MSD curve is somewhat linear for the disordered states.

### Pressure Profiles

We also calculated the local pressure tensor (due to particle interactions), *p*(*z*), along the field direction (*z*-axis) using the definition due to Kirkwood and Buff:^38−40^
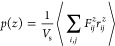
3The system was divided into small slices parallel to the *x*–*y* plane with *V*_s_ being the volume of each slice. For every particle *i* in a given slice centered at *z* (with thickness δ*z*), we calculated the component of the virial in the *z* direction, *F*_*ij*_^*z*^*r*_*ij*_^*z*^, by summing the pairwise force with every other particle, *j*. This, in turn, is summed over every particle in the slice. The bracket ⟨⟩ represents a specific average over the trajectory (as described below). An average over all the slices in the simulation box gives the total virial pressure tensor of the system. The ideal gas contribution to the pressure is much smaller than the virial contribution, and we neglect it.

**Figure 4:**
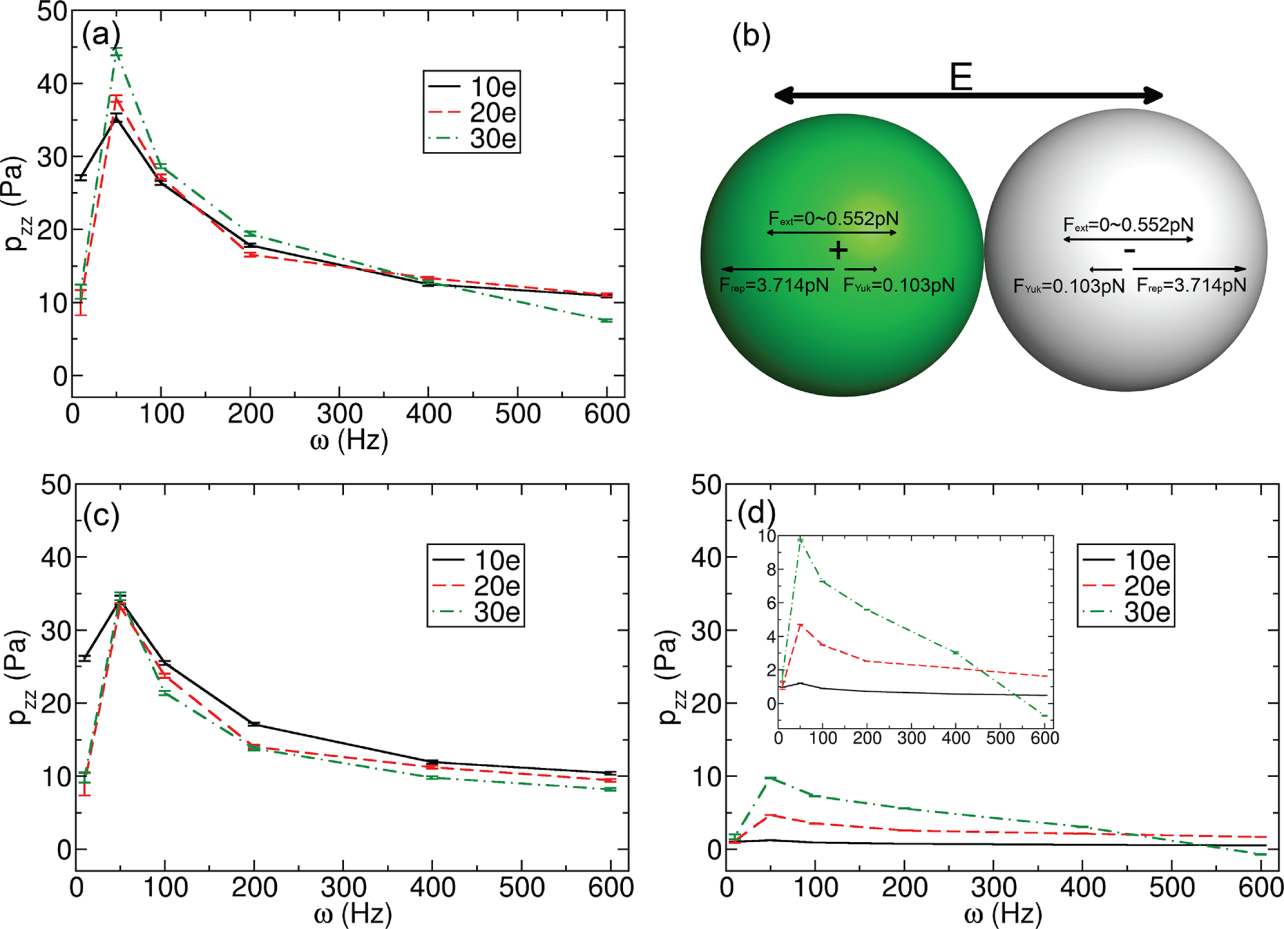
(a) Relationship between equilibrium virial pressure tensors *p*_*zz*_ and ω values for systems with various particle charges. (b) Schematic diagram for the force decomposition of ±20*e* charged particles. (c,d) Contributions from steric repulsions and electrostatic interactions to *p*_*zz*_, respectively. The enlarged inset of the contribution from electrostatic interactions to *p*_*zz*_ is shown for clarify.

In Figure 4a, we show the average total virial pressure, calculated at the end of each oscillating period of the simulated trajectory (after steady state has been reached). Here, the maximum applied force was kept constant at *F*_0_ = 0.552 pN (as described in Figure 1e). The resulting total average pressure (denoted by *p*_*zz*_) is plotted against the field frequency, ω. The relative magnitudes of the repulsive and Yukawa contributions to the virial can be appreciated by choosing a pair of particles at contact (with separation *r* = σ = 150 nm). Detailed information on these potentials are provided in the SI. In Figure 4b, we consider such a pair for the case, ±20*e*. At this separation, the steric term is much greater than the Yukawa contribution. The average steric and Yukuwa contributions to the virial *p*_*zz*_ are given in Figure 4c,d.^41,42^ As expected, the short-ranged steric repulsions dominate the virial pressure over the range of ω considered, at least for these colloidal charges.

Over most of the frequency range (50–600 Hz), the system adopts the jammed steady-state form where *p*_*zz*_ generally decreases with large ω. At smaller ω, where the time period over which particles in the jammed phases are forced against each other is longer, the pressure in the compressed bands will increase. This increase in *p*_*zz*_ with decreasing ω reaches a threshold value (located at ω ≈ 50 Hz). Below this threshold, the system is unable to maintain the perpendicular bands of the jammed structure and the steady state adopts a different form, with a significant decrease in *p*_*zz*_. At ω = 10 Hz, particles with charge ±20*e* and ±30*e* form laned structures. For particles with ±10*e*, lanes do not appear explicitly, and the order parameter, Ψ, is reduced but not zero, indicating mixing of particles with opposite charges and perhaps some incipient laning. The maximum value of *p*_*zz*_, at which phase change occurs, is similar for all of the systems investigated (almost identical steric contributions). This indicates the possible occurrence of an instability in the jammed phase, wherein parallel bands of like-charged particles are unable to remain perpendicular to each other beyond a threshold value of the perpendicular pressure. Instead, at or beyond this threshold value, the surfaces of the bands undergo significant buckling *via* concerted motions that cause the bands to penetrate one another. This ultimately gives rise to laned steady states, at least at the higher charges considered (and most likely at the lowest charge, as well).

**Figure 5:**
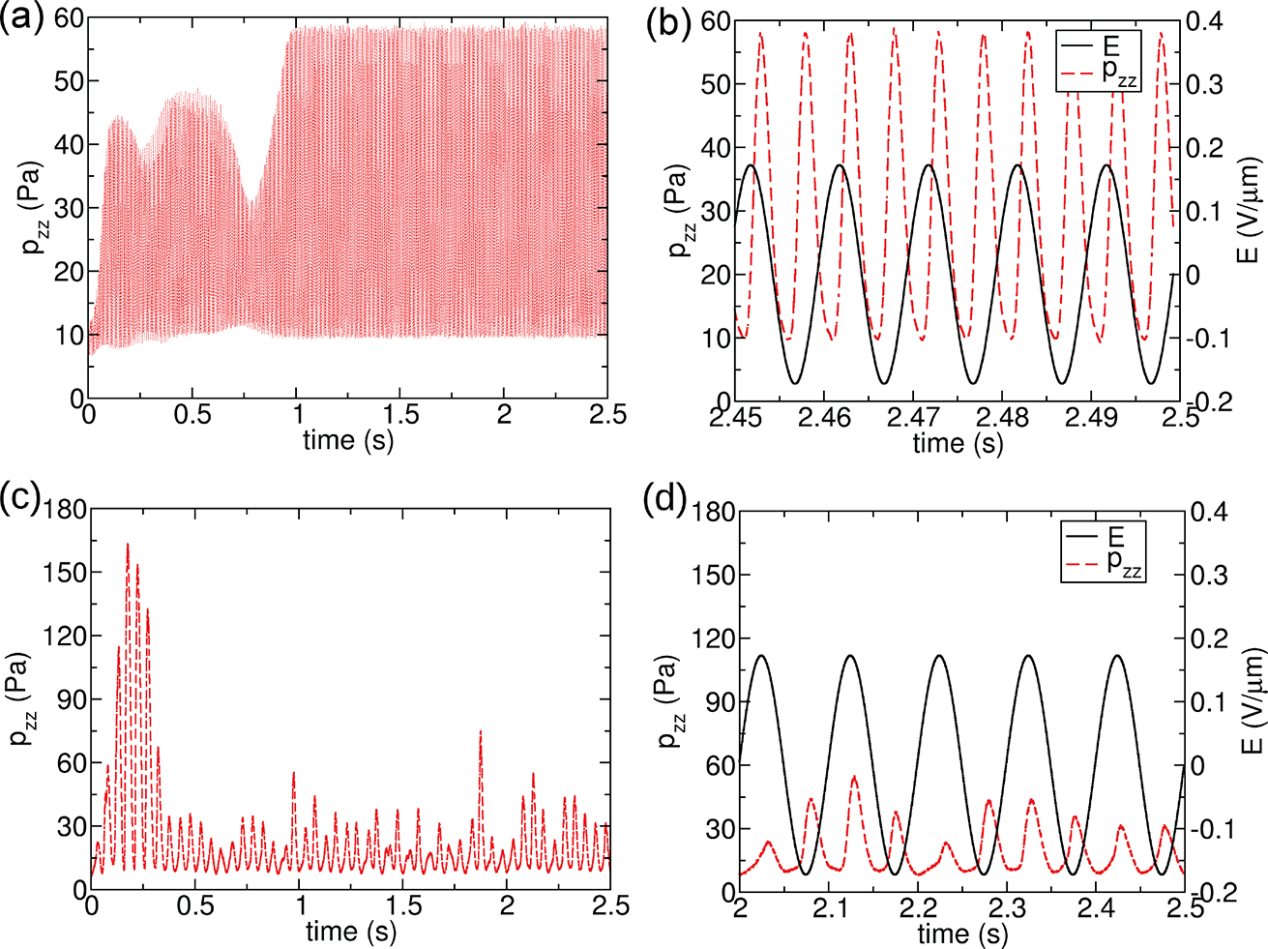
Time evolutions of pressure profiles along the field direction, at different oscillating periods, for the particles with *q* = ± 20*e*. (a,b) Results at 100 Hz, and (c,d) are at 10 Hz. (b,d) Last five oscillating periods of *p*_*zz*_, with the AC fields shown as black curves for comparison, as well as the *y* axis on the right-hand side in each graph.

**Figure 6:**
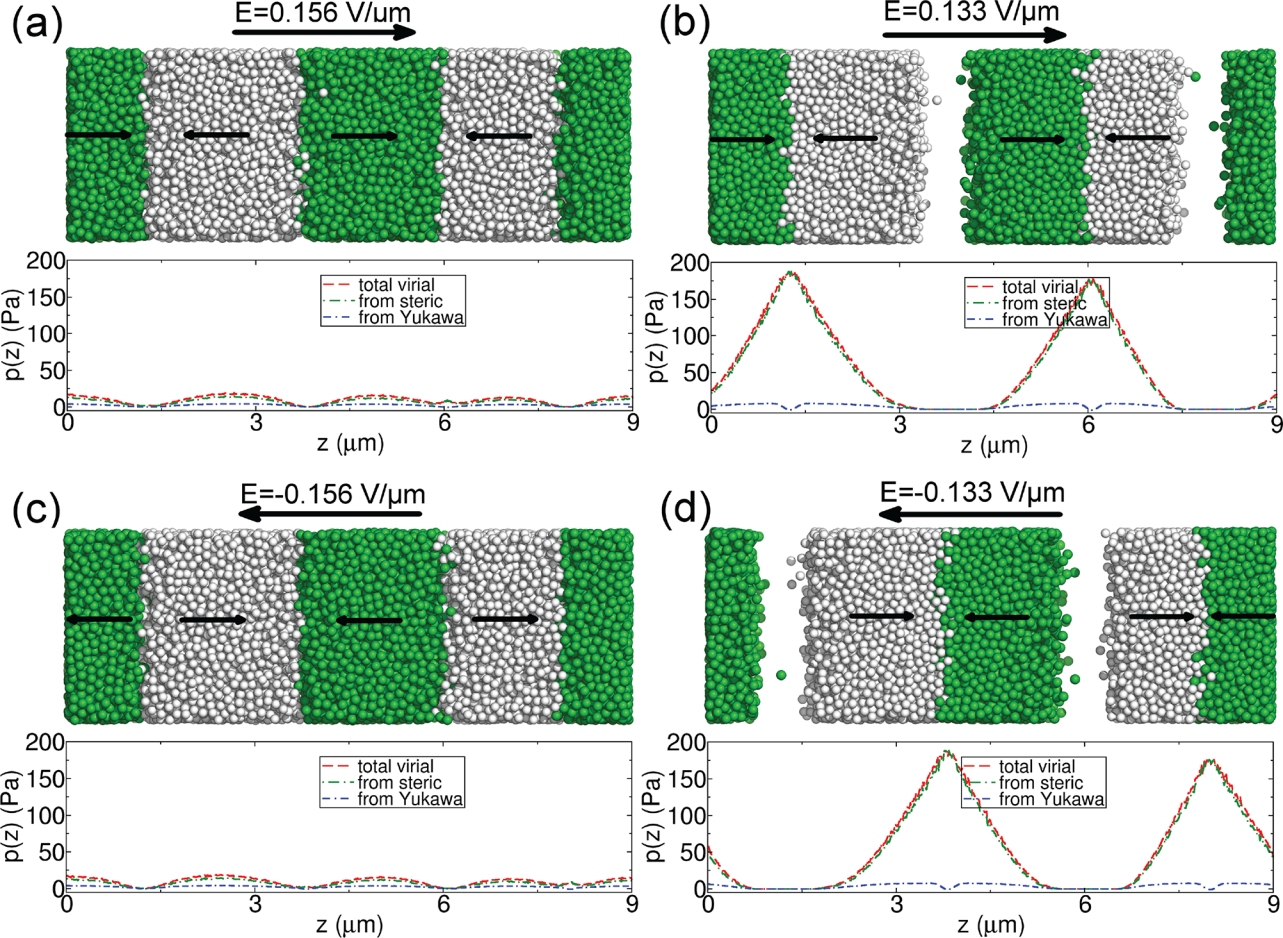
Pressure profiles, *p*(*z*), along the AC field direction, under ω = 100 Hz, evaluated at times which correspond to the minima and maxima of *p*_*zz*_ during an oscillating period. The instantaneous AC field strength is shown at the top of each figure, with the field direction indicated by an arrow. The snapshots are placed by aligning with the pressure profiles. The arrows in the bands illustrate the directions of the forces induced by the AC fields.

Figure 5 shows the total virial pressure tensor, *p*_*zz*_(*t*), resolved as a function of time, for the frequencies ω = 100 and 10 Hz. These are the same systems displayed in Figure 2. We see that *p*_*zz*_ oscillates in response to the applied field with a magnitude that appears to be commensurate with the order parameter Ψ. For example, in Figure 5a, in the first 0.1 s, the amplitude of *p*_*zz*_(*t*) increases during phase separation process and then decreases until about 0.3 s. This is the same behavior seen for the order parameter, Ψ, in this system (see Figure 2a). The amplitude of *p*_*zz*_ also has peaks at about 0.5 and 0.75 s, which are consistent with Ψ. The magnitude of the pressure tensor is thus directly correlated with the degree of ordering, wherein large domains of like-charged particles exert forces on each other as they respond in opposite ways to the applied field. After the pressure amplitudes reached a steady state, we plotted the last five oscillating periods of *p*_*zz*_(*t*) together with the AC field (see Figure 5b). The oscillating frequencies of *p*_*zz*_ are twice as high as the AC field frequencies for ω = 100 Hz, and the phase seems to be somewhat delayed. As expected, there are two maxima in *p*_*zz*_ within an oscillation period of the field, as the particles respond to both positive and negative values of the field. To more clearly show this, we have also considered the spatially resolved profile, *p*(*z*), at specific times in a single period, as shown in Figure 6. The *p*(*z*) profiles are small in magnitude and relatively flat at the same time when the total *p*_*zz*_ is at its minimum (Figure 6a,c). On the other hand, *p*(*z*), has larger distinct peaks at the collision interfaces when *p*_*zz*_ is at its maximum (Figure 6b,d). The contributions from the Yukawa potential to *p*(*z*) are much smaller than the steric repulsion, especially when *p*_*zz*_ reaches the maximum values. In addition, there are small minima in the Yukawa contribution to *p*(*z*) at the collision interfaces, due to the electrostatic attraction between the bands with oppositely charged particles.

In the system with ω = 10 Hz, the *p*_*zz*_ values increase rapidly to about 160 Pa when the jammed structure forms at 0.177 s (Figure 5c and Figure 2b), such a large internal pressure makes the jammed structure unstable, and bands penetrate each other and eventually form lanes. After the penetration process finishes, at approximately 0.3 s, the *p*_*zz*_ values decrease quickly. We still find that *p*_*zz*_ fluctuates with the AC field, due to the nonuniform laned structure, as well as some partial and short-lived jammed band structures formed at the maximum of absolute electric field. The *p*(*z*) profiles at the maximum and minimum values of *p*_*zz*_ under 10 Hz in an oscillating period are shown in Figure S6. The *p*(*z*) profiles show peaks and wells at the maxima of *p*_*zz*_ in an oscillating period, and they are flat at the minima of *p*_*zz*_.

### Entropy Production

The systems investigated here, driven by AC fields, dissipate heat to the implicit reservoir *via* the overdamped Langevin dynamics. The dynamics, being nonreversible, also produce entropy in the system, which at steady state is matched by the entropy dissipated *via* the heat loss to the reservoir. From the first law of thermodynamics, the heat dissipated is also equal to the work performed by the external force under steady-state conditions. The heat dissipation rate is given by ,^43−46^ where **ṙ**_*i*_ is the velocity of particle *i*, γ is the friction coefficient, ξ_*i*_ is the random force in the overdamped Langevin equation and “·” is a Stratonovich product. The averaged dissipation rate for the system over an oscillating period τ_0_ is
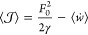
4The derivation of this equation is provided in SI. The first term on the right-hand side corresponds to the contribution from the applied force, which has an amplitude *F*_0_. It is the work performed on *ideal* particles by the applied force. The second term accounts for contributions from particle interactions and is given by^3,46,47^
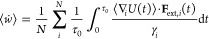
5where *U*(*t*) is the nonbonded interaction between particles (steric repulsion and Yukawa). The quantity ⟨*ẇ*⟩ can be thought of as the work done by the restoring forces generated by the colloidal particles, as a consequence of them responding to the applied field.

**Figure 7:**
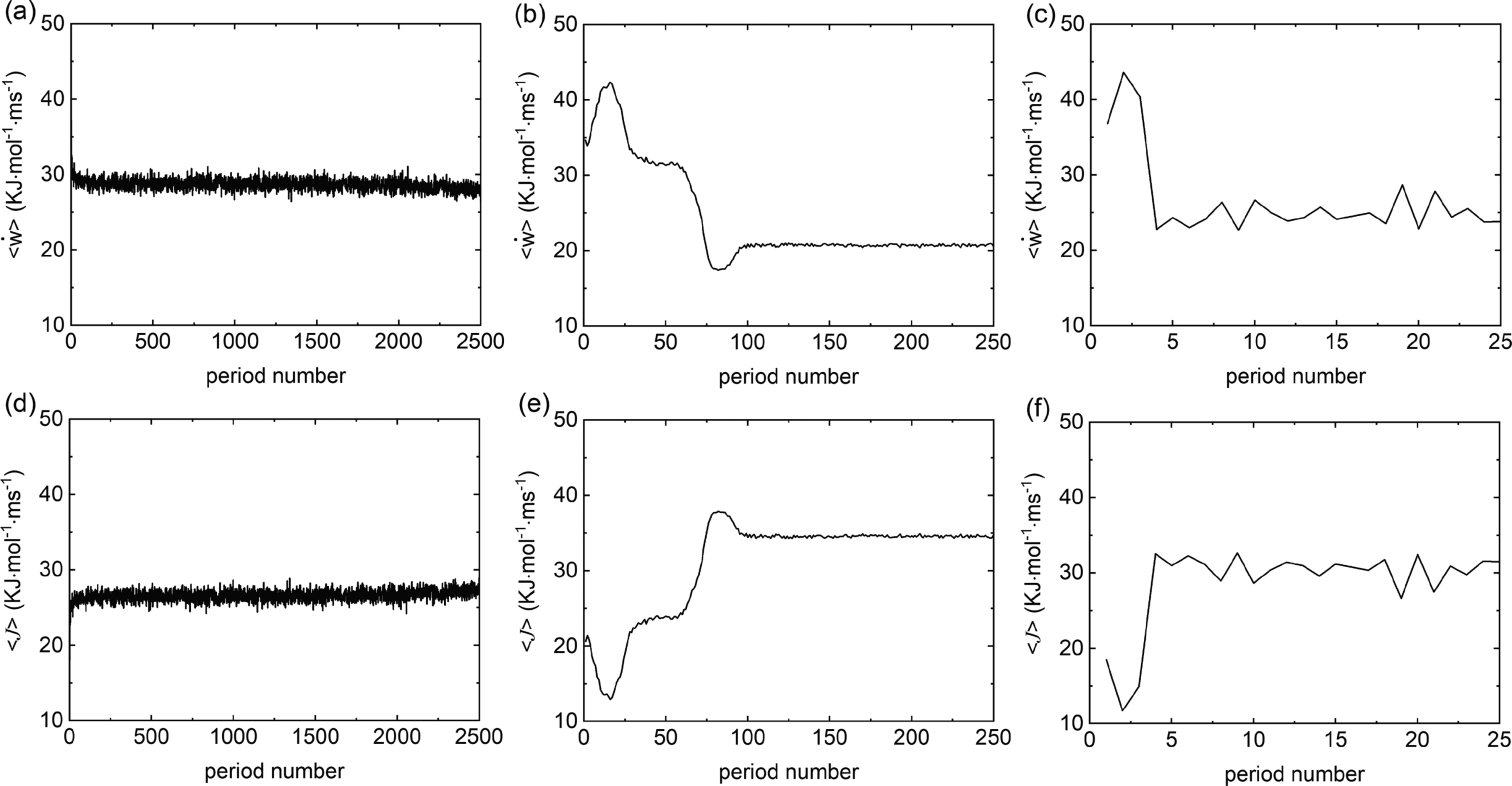
Relationship between ⟨*ẇ*⟩ (a–c), (d–f), and oscillating period serial number for ±20*e* charged particle systems. (a,d) Results under 1 kHz. (b,e) Results under 100 Hz. (c,f) Results under 10 Hz.

The values of ⟨*ẇ*⟩ at different oscillating periods for the ±20*e* charged particle systems are displayed in Figure 7 (and in Figure S7, in order to provide more comprehensive view for the time evolution of the rate of work). At 1 kHz (Figure 7a,d), ⟨*ẇ*⟩ and hence  do not change significantly during the whole simulation, implying that the (initial) disordered structure is also the stable steady state. At 100 Hz (Figure 7b,e), ⟨*ẇ*⟩ increases until about the 15th period. This increase in work rate corresponds to the initial ordering of the fluid and significant collisions between oppositely charged particles. We then find that ⟨*ẇ*⟩ subsequently reduces, coinciding with a decrease in the area of the collisional interfaces between oppositely charged particles as bands begin to form. There is a minimum for ⟨*ẇ*⟩ at roughly the 75th period, due to the penetration of bands, as seen at 0.77 s in Figure 2a, in order to form thicker layers. The rate of work remains essentially constant after the 100th period. Here, the system has reached the steady state, which is a jammed structure, and the heat dissipation rate is also constant. It appears that ⟨*ẇ*⟩ decreases as the system moves from the uniform (random) initial configuration to the jammed steady state, suggesting that the latter corresponds to a condition of least work being performed by the restoring forces of the colloidal particles per oscillation period. This is equivalent to a maximum rate of dissipation of heat to the surrounds or maximal entropy production rate.^44,48^ A similar analysis for the system where the applied force oscillates at ω = 10 Hz (Figure 7c) shows that ⟨*ẇ*⟩ increases initially, similar to Figure 7b, then decreases again with the establishment of the steady-state laned structure.

As ⟨*ẇ*⟩ describes the work done by the restoring forces in the fluid, the nonbonding interactions between the colloids play an important role. At lower frequency, collisions between oppositely charge particles occur over a longer time within each period, which also leads to the possibility of the fluid restructuring, in order to minimize the prevalence of repulsive forces. This follows from the simple expectation that fluid particles will follow the “path of least resistance” under the action of the external force, and if given time, the fluid may restructure in order to achieve this. Therefore, we see emergence of jammed and laned phases at the lower frequencies. On the other hand, if the external force is disrupted, *e.g.*, changes direction too often, the fluid does not have sufficient time for ordering. Thus, at 1 kHz, the fluid remains uniformly disordered and colloidal particles frequently collide with several others of opposite charge. Although the collisions between particles at 100 Hz give rise to stronger repulsive forces than at 1 kHz, the jammed band structure has a lower value of ⟨*ẇ*⟩. This is because collisions in the jammed structure occur mainly between particles at or near the interfaces and thus occur much less often than in the disordered state at 1 kHz. The arguments above suggest we may expect that ⟨*ẇ*⟩ will decrease with decreasing frequency. Interestingly, ⟨*ẇ*⟩ at steady state (Figure 7a–c) shows a nonmonotonic behavior *versus* field frequencies. For instance, ⟨*ẇ*⟩ is largest at 1 kHz, almost 30 kJ·mol^–1^·ms^–1^, whereas at 100 Hz, it is somewhat smaller than at 10 Hz. We hypothesized that, given the geometry of the laned structures, with only a single central lane of one type of particle surrounded by the other type, there may be a size-dependent effect. Thus, we investigated the system under 10 Hz with a simulation box width (in the *x* and *y* directions) scaled by the factor λ (=2). The density profiles of the system are shown in Figure S5d,f, which displays again a central lane of one type of particle surrounded by the opposite type of particles, similar to the original simulation (λ = 1). As the main contribution to the work comes from the interfaces between the lanes (which scale linearly with λ), ⟨*ẇ*⟩, which is a per particle quantity (the number of particles scaling with λ^2^), we expect, and see, a decrease in ⟨*ẇ*⟩ compared with the smaller simulation (Figures S8 and S9). For both the geometry of the uniform and jammed steady states, we do not expect to see such a significant size dependence. Compared to the other results, we now see a monotonic behavior with respect to frequency.

**Figure 8:**
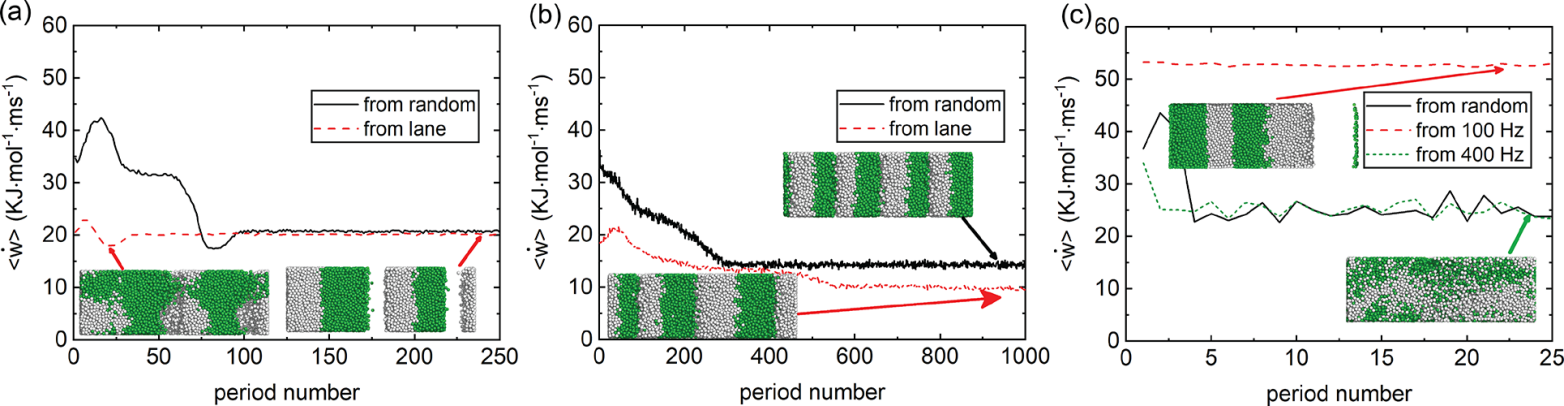
Relationship between ⟨*ẇ*⟩ and oscillating period serial number for ±20*e* charged particle systems. (a,b) Values of ⟨*ẇ*⟩ in different oscillating periods under ω = 100 and 400 Hz, respectively; the initial configuration is from the laned structure obtained under 10 Hz (red curves), as well as the comparison with the results from random disordered initial configurations (black curves). (c) Values of ⟨*ẇ*⟩ under 10 Hz using the initial configurations of jammed band structures from 100 and 400 Hz, as well as random disordered state.

In an effort to further elucidate the guiding principles that dictate the formation of steady-state structures, we compared the behavior of ⟨*ẇ*⟩ for various laned and jammed structures under those conditions where they *do not* appear to be the preferred steady state. For instance, the laned structure formed under 10 Hz was used as the initial configuration for the simulation at 100 Hz in order to determine if the laned structure transitions to the apparently preferred jammed structure (which forms spontaneously from a random initial configuration) or *vice versa*. The rates of work ⟨*ẇ*⟩ for the simulations with varying frequencies as well as some representative snapshots are shown in Figure 8. The laned structures obtained under 10 Hz did indeed transition to the jammed structure at ω = 100 and 400 Hz (see Figure 8a,b). Furthermore, as the simulation progressed in all cases, there was a general progression of the system to lower ⟨*ẇ*⟩, *i.e.*, to maximize the rate of entropy production. This can be understood as a tendency of the system to respond in a way to minimize the restoring forces in the fluid. In the initial laned structure, the sum of collisional forces between the particles is generally smaller than those in the random disordered state, hence, ⟨*ẇ*⟩ for the laned steady states at the beginning of the simulations is lower than that with a random state. However, both kinds of initial states find their way to the jammed states (with an even lower value for ⟨*ẇ*⟩). In addition, the steady-state value of ⟨*ẇ*⟩ at 400 Hz, initiated from the laned structure, is lower than that emerging from the random disordered state. This is because of the presence of fewer bands in the latter case (snapshots in Figure 8b). This perseverance of two jammed states with different rates of entropy production suggest the possibility of metastable steady states in these driven systems.

We also initiated a simulation at ω = 10 Hz with jammed steady-state structures formed at 400 and 100 Hz. The initial jammed state (from the 400 Hz simulation) did transition to the laned structure appropriate to ω = 10 Hz (snapshot with green arrow in Figure 8c). Again, there was a concomitant decrease in ⟨*ẇ*⟩ during this transition (Figure 8c). On the other hand, the initial jammed state formed at 100 Hz remains (meta-)stable with a higher value for ⟨*ẇ*⟩, indicating a metastable steady state. It seems that the jammed structure formed under 100 Hz is too stable to be penetrated, even this low frequency, ω = 10 Hz.

While our results thus suggest that the driven system appears to adopt a steady state of maximum entropy production, a general proof of this principle is still lacking. In this context, it is relevant to note that Schmidt and co-workers have suggested that lane formation can be understood in terms of so-called superadiabatic forces, generated by the dynamics of a driven fluid.^49,50^

## Conclusions

In summary, we study oppositely charged colloidal particles using overdamped Langevin simulations. Under an applied AC field, they form jammed bands at intermediate frequencies and lanes under lower frequencies. In the jammed band phases, under a constant force amplitude, the system displays a high-order parameter, especially for systems with a rather low particle charge. The pathway for generating jammed bands is a stepwise mechanism. First, metastable thin bands are obtained, which then penetrate and merge to generate the thicker bands. The formation of laned structures also occurs *via* intermediate jammed state in some systems. The tendency to form jammed bands is larger under lower frequency, in the regime below 50 Hz. This is due to faster diffusion along the field direction, as well as stronger collisions between adjacent bands. The virial pressures along the field direction show similar nonmonotonic frequency dependencies, as the contributions from steric repulsion between the particles play a major role in the virial pressures. The oscillation period for the pressure profiles is half of the AC field because of two sets of collisions between particles in a period of AC field. The decreasing rate of work done by the restoring forces indicates a tendency to minimize collisional interfaces giving rise to restoring forces, which oppose the influence of the applied field. The result is to increase heat dissipation to the surrounding universe, leading to maximum entropy production.

The technical approaches and results obtained in this work provide some possibility for future investigations. For example, colloidal polarizability and field-induced anisotropic distributions of the small ions that surround the particles would be interesting aspects to consider in future work.^51^ Although we have provided the basic underlying mechanisms for phase transitions of colloidal particles under AC fields with our model, we envisage that a deeper physical insight would be obtained if dipolar effects are considered. In addition, it would furthermore be worthwhile to extend the scope to systems with other particle shapes and also consider particle softness.

## Models and Methods

We implement an effective AC electric field of the form **E**(*t*) = *E*_0_ sin(2πω*t*), where *E*_0_ is the maximum electric field strength. The electric field is chosen to be uniform and directed along a particular axis of the simulation box. The field influences the dynamics by the subsequent force on the colloidal particles, which was assumed to have the magnitude, **F**_ext_(*t*) = **E**(*t*)*q* (*q* is the particle charge), and acts in the field direction. The field frequency ω is the reciprocal of the oscillation period τ_0_, ranging between 10 Hz and 1 kHz.

The charged colloidal particle is represented by a single charged sphere model. Details of the model in our simulations are provided in SI. The *effective* particle diameter σ is set to 150 nm in real units. We use a 3D simulation box with *L*_*x*_ = *L*_*y*_ = 3 μm and *L*_*z*_ = 9 μm, and the external field acts along *z* direction. Periodic boundary conditions are applied in all three dimensions. Altogether, we use 19200 colloidal particles in the system. In any one system, we use equal numbers of positive and negative particles, with identical absolute charges, and the particle charges vary from ±10*e* to ±50*e*. The Bjerrum length λ_B_ is set to 10 nm in order to mimic an organic solvent, and the Debye length λ_D_ = 20 nm reflects the screening of smaller ions. The total simulation time is about 2.5 s in real units.

All of the simulations are performed with the software package LAMMPS.^52^ The particles are assumed to be dispersed in a highly viscous solvent, so that their dynamics are overdamped. The overdamped Langevin dynamics method has been implemented in LAMMPS, as described in the SI. We keep the solvent viscosity to be approximately 1.17 cP in the systems we study.

## Supporting Information Available

Detailed description of charged colloidal particle model, overdamped Langevin dynamics method, derivation of heat dissipation and rate of work equations, additional snapshots and time evolutions of order parameters; the results of larger simulation systems and system size effects on phase transition pathways and density profiles; spatial pressure profiles in lane structure; the results of work and heat dissipation, including full time evolutions, the values in system with larger box width, as well as in very ordered lane structures (PDF)
Trajectory movie for the phase transition of ±20*e* charged colloidal particles under AC field with a frequency of 100 Hz, corresponding to Figure 2a (MP4)
Trajectory movie for phase transition dynamics of ±20*e* charged colloidal particles under AC field with 10 Hz, corresponding to Figure 2b (MP4)
