# Supplementary material for: Phase Transitions of Oppositely Charged Colloidal Particles Driven by Alternating Current Electric Field
Source: ACS Nano. 2021 Feb 12;15(2):2363–73. doi: 10.1021/acsnano.0c04095 (PMC8023798; doi:10.1021/acsnano.0c04095)
Supplement: Supplementary file 1 — nn0c04095_si_001.pdf [file nn0c04095_si_001.pdf]

Supporting Information for: Phase Transitions of Oppositely Charged Colloidal Particles Driven by Alternating Current Electric Field

Bin Li,[a*,†,‡,¶] Yong-Lei Wang[a],[§] Guang Shi,[‖] Yangyang Gao,[⊥,#] Xinghua Shi,[†] Clifford E. Woodward,[*,@] and Jan Forsman[*,‡]

†*Laboratory of Theoretical and Computational Nanoscience, CAS Key Laboratory for Nanosystem and Hierarchy Fabrication, CAS Center for Excellence in Nanoscience, National Center for Nanoscience and Technology, Chinese Academy of Sciences, Beijing 100190, China*

‡*Theoretical Chemistry, Chemical Center, Lund University, P.O. Box 124, S-221 00 Lund, Sweden*

¶*School of Chemical Engineering and Technology, Sun Yat-sen University, Zhuhai 519082, China*

§*Department of Materials and Environmental Chemistry, Arrhenius Laboratory, Stockholm University, SE-106 91 Stockholm, Sweden*

‖*Department of Chemistry, University of Texas at Austin, TX, Austin, 78712, USA*

⊥*Key Laboratory of Beijing City on Preparation and Processing of Novel Polymer Materials, Beijing University of Chemical Technology, 10029, People's Republic of China*

#*State Key Laboratory of Organic-Inorganic Composites, Beijing University of Chemical Technology, 10029, People's Republic of China*

@*School of Physical, Environmental and Mathematical Sciences, University College, University of New South Wales, ADFA, Canberra ACT 2600, Australia*

E-mail: libin76@mail.sysu.edu.cn; c.woodward@adfa.edu.au; jan.forsman@teokem.lu.se

[a]B. L. and Y.-L. W. contributed equally to the work.

Models and Methods

Charged Colloidal Particle Model

We adopt a smooth repulsive Mie potential developed by Jover and co-workers,[1] to reflect the steric repulsion between colloidal particles, which aided our molecular dynamics (MD) simulations before.[2] The Mie potential is given by

$$U(r) = \frac{\lambda_r}{\lambda_r - \lambda_a}(\frac{\lambda_r}{\lambda_a})^{\frac{\lambda_a}{\lambda_r-\lambda_a}}\epsilon[(\frac{\sigma}{r})^{\lambda_r} - (\frac{\sigma}{r})^{\lambda_a}] \tag{1}$$

where $\lambda_r = 50$ and $\lambda_a = 49$. The potential is truncated and shifted at its minimum, which results in the purely repulsive pseudo hard sphere potential:

$$U_{(50,49)}(r) = \begin{cases} 50(\frac{50}{49})^{49}\epsilon[(\frac{\sigma}{r})^{50} - (\frac{\sigma}{r})^{49}] + \epsilon, & r < \frac{50}{49}\sigma \\ 0, & r \geq \frac{50}{49}\sigma \end{cases} \tag{2}$$

in which ϵ is the interaction parameter, set at 1 $k_\mathrm{B}T$, and k_B is Boltzmann constant. The quantity, σ, represents the *effective* particle diameter, and is set to 150 nm in real unit.

The electrostatic interaction between the colloidal particles is modeled using the screened Coulomb, or Yukawa potential,[3,4]

$$U_e(r) = \frac{q_i q_j \lambda_\mathrm{B}}{(1+\kappa\sigma/2)^2}\frac{e^{-\kappa(r-\sigma)}}{r} \tag{3}$$

where q_i, q_j are the charges of particles i and j, respectively. $\lambda_\mathrm{B} = e^2/(4\pi\epsilon_0\epsilon_r k_\mathrm{B}T)$ is the Bjerrum length. This is set to 10 nm, which corresponds to the dielectric constant $\epsilon_r \approx 5.6$ and mimics an organic solvent. We also have $\kappa^{-1} = \lambda_\mathrm{D} = (4\pi\lambda_B \sum_{i=1}^{N} n_i z_i^2)^{-1/2}$, which is the so-called Debye length. This Yukawa potential accounts for the screening of the electrostatic colloid interactions by the presence of smaller ions in solution. We set $\kappa\sigma = 7.5$, which means $\kappa^{-1} = 20$ nm in this work.

Overdamped Langevin Dynamics

The overdamped Langevin dynamics method is implemented in LAMMPS, which neglects inertia of particles. The equation of motion for particle i is expressed as,

$$\dot{\mathbf{r}}_i = \frac{1}{\gamma_i}\left(-\nabla_i U + \mathbf{F}_{ext}\right) + \xi_i \tag{4}$$

where U represents the total particle interaction including Mie and Yukawa potentials, $\mathbf{F}_{ext} = \mathbf{E}q = E_0 \sin(2\pi\omega t)$ is the time-dependent external force by the electric field, $\gamma_i = 3\pi\eta\sigma$ is the friction coefficient, according to Stokes' Law and η is the solvent viscosity, which we choose to be approximately 1.17 cP, ξ_i is a Gaussian random noise with zero mean and unit variance. The Brownian time $\tau = \sigma^2/D$ is used as time unit, where $D = k_{\mathrm{B}}T/\gamma$ is the bare diffusion coefficient of a Brownian particle, the time-step for integrations $\Delta t = 3.73 \times 10^{-6}\tau$.

Heat Dissipation and Rate of Work

The averaged heat dissipation rate is described as $\mathcal{J} = \langle \dot{\mathbf{r}}_i \cdot (\gamma\dot{\mathbf{r}}_i - \xi_i)\rangle$, it is also shown in the main article. We use $\dot{\mathbf{r}}_i$ in Supporting equation 4, to substitute $\dot{\mathbf{r}}_i$ in the equation, and neglect the change rate of averaged internal energy $dU/dt = \langle \dot{\mathbf{r}}_i \cdot \mathbf{F}_{c,i}\rangle$, as dU/dt is much smaller than the rate of work $\langle \dot{w}\rangle$ and dissipation $\mathcal{J}$ in our systems. Thus we obtain the form for $\mathcal{J}$ as[5]

$$\mathcal{J} = \langle \dot{\mathbf{r}}_i \cdot \mathbf{F}_{ext,i}\rangle \tag{5}$$

The $\dot{\mathbf{r}}_i$ in Supporting equation 5 is replaced by its expression in Supporting equation 4 again, after that we calculate the heat dissipation over an oscillating period τ_0, then we obtain

$$\langle \mathcal{J}\rangle = \frac{1}{N}\sum_i^N \frac{1}{\tau_0}\int_0^{\tau_0} \langle \frac{\mathbf{F}_{ext,i}\cdot\mathbf{F}_{ext,i}}{\gamma} - \frac{\nabla_i U \cdot \mathbf{F}_{ext,i}}{\gamma}\rangle dt \tag{6}$$

the first term on the right-hand side corresponds to the contribution from external force, or the work of external force on *ideal* particle, the second term means the contribution from

particle interactions. Then we substitute $\mathbf{F}_{ext,i}$ with the expression $\mathbf{F}_{ext,i} = F_0 \sin(2\pi\omega t)$, and get a simplified expression for $\langle \mathcal{J} \rangle$

$$\langle \mathcal{J} \rangle = \frac{F_0^2}{2\gamma} - \langle \dot{w} \rangle \tag{7}$$

which is also shown as equation 4 in the main article. The expression is similar as the heat dissipation rate calculated by Tociu and co-workers,[5] but as we adopt the sine-wave external force *versus* time, the first term contributed by the external forces is slightly different.

References

1. Jover, J.; Haslam, A. J.; Galindo, A.; Jackson, G.; Müller, E. A. Pseudo Hard-Sphere Potential for Use in Continuous Molecular-Dynamics Simulation of Spherical and Chain Molecules. *J. Chem. Phys.* **2012**, *137*, 144505.

2. Lu, H.; Li, B.; Nordholm, S.; Woodward, C. E.; Forsman, J. Ion Pairing and Phase Behaviour of an Asymmetric Restricted Primitive Model of Ionic Liquids. *J. Chem. Phys.* **2016**, *145*, 234510.

3. Denton, A. R.; Löwen, H. Stability of Colloidal Quasicrystals. *Phys. Rev. Lett.* **1998**, *81*, 469–472.

4. Vissers, T.; Wysocki, A.; Rex, M.; Löwen, H.; Royall, C. P.; Imhof, A.; van Blaaderen, A. Lane Formation in Driven Mixtures of Oppositely Charged Colloids. *Soft Matter* **2011**, *7*, 2352–2356.

5. Tociu, L.; Fodor, É.; Nemoto, T.; Vaikuntanathan, S. How Dissipation Constrains Fluctuations in Nonequilibrium Liquids: Diffusion, Structure, and Biased Interactions. *Phys. Rev. X* **2019**, *9*, 041026.

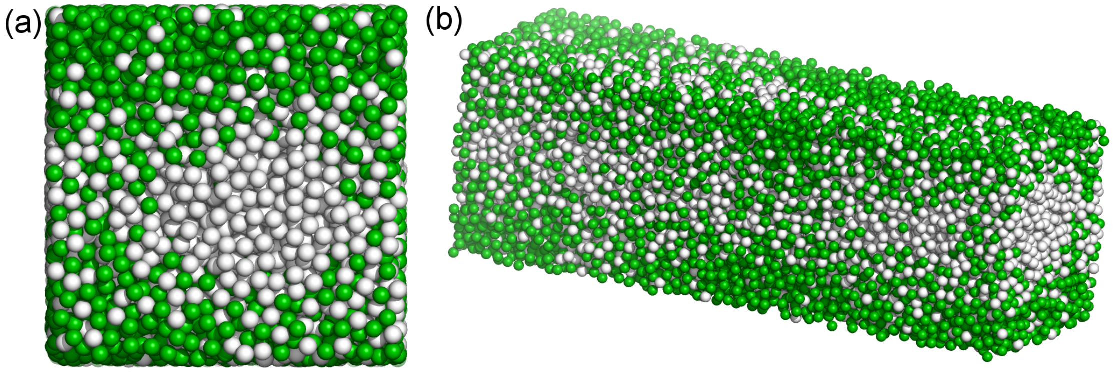

Figure S1: The snapshots of $\pm 40e$ under the AC field with $E_0 = 0.115$ V/μm and $\omega = 10$ Hz. (a) View with the top direction. (b) View with tilted direction.

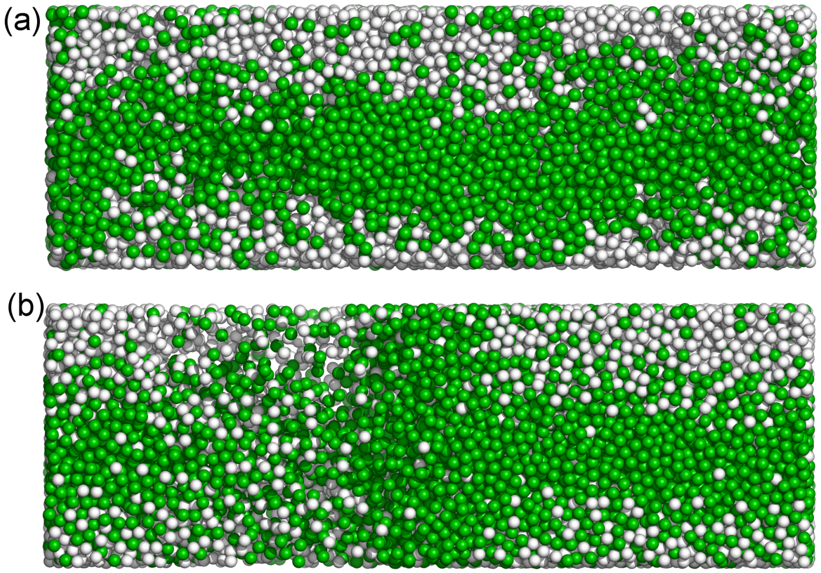

Figure S2: The snapshots of charged colloidal particles under DC field with $E_c = E_0/\sqrt{2}$, corresponding to the constant external force $F_c = F_0/\sqrt{2} = 0.39$ pN. (a) Result of $\pm 10e$ charged colloidal particles, (b) Result of $\pm 50e$ charged colloidal particles.

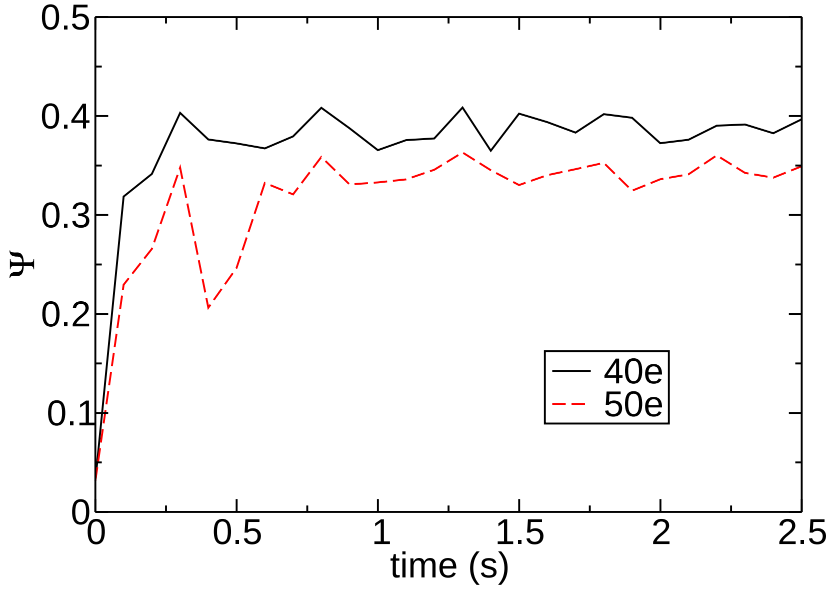

Figure S3: The time evolutions of Ψ values for $\pm 40e$ and $\pm 50e$ charged colloidal particles under $\omega = 10$ Hz.

System Size Effect

We check the influence of size effect on the phase transition pathways for the two systems shown in Figure 2, by elongating the simulation box along field direction for generating jammed band, and increasing the box width perpendicular to field for lane formation. Both of the two larger boxes are increased by a factor $\lambda = 2$ in z direction, and x, y directions, respectively. The similar pathways could also be observed, which are shown in Figure S4.

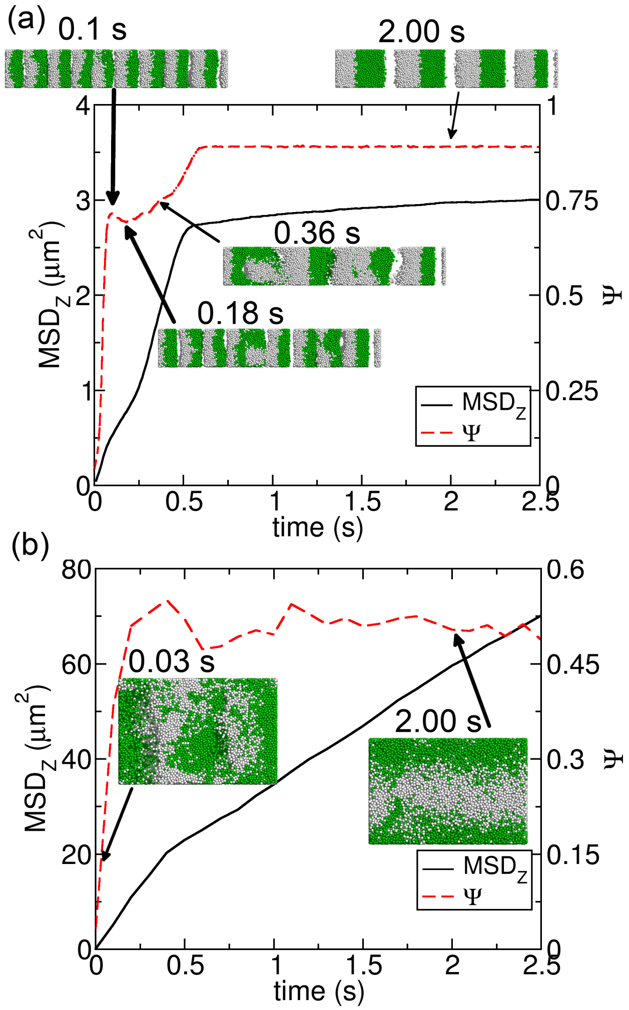

Figure S4: Time evolutions of MSD along the AC field direction and Ψ for systems containing particles with $\pm 20e$ in larged systems, at an AC field strength of $E_0 = 0.1725$ V/μm, by considering the system size effect. The snapshots are taken at the simulation times which are indicated by arrows. (a) $\omega = 100$ Hz; (b) $\omega = 10$ Hz.

Density Profiles

We plot the number density profiles of positively charged particles along the electric field of the two systems in Figure 2, after the systems reach steady state ($t \geq 2$ s). We choose the density profiles at 4 moments, when the field strength is E_0, 0, $-E_0$ and 0 in different periods, corresponding the simulation times are $(n + \pi/2)\tau_0$, $(n + \pi)\tau_0$, $(n + 3\pi/2)\tau_0$ and $n\tau_0$, respectively. The results are displayed in Figure S5. The local oscillations of jammed particles could also be observed *via* the different profiles of densities (Figure S5a), and the lanes formed in $\pm 20e$ charged particles under 10 Hz are not so perfect, according to the density fluctuations in Figure S5b. We also check the influence of system size effect on the density profiles, and find that the elongating simulation system under 100 Hz shows similar density profile (Figure S5c) as Figure S5a, and the density profile seems to be flatter in the broadening system under 10 Hz (Figure S5d). Then we calculate the densities at the end of each period in x-y dimension perpendicular to the field for the laned structure. The contour plot of the x-y densities is shown in Figure S5e, and the result for the broadening system under 10 Hz is shown in Figure S5f. The x-y densities show a flattened square profile in the smaller simulation system, and band profile in the broadening system. The system size effect has no influence on the jamming band formation, but influences the lane formation a little.

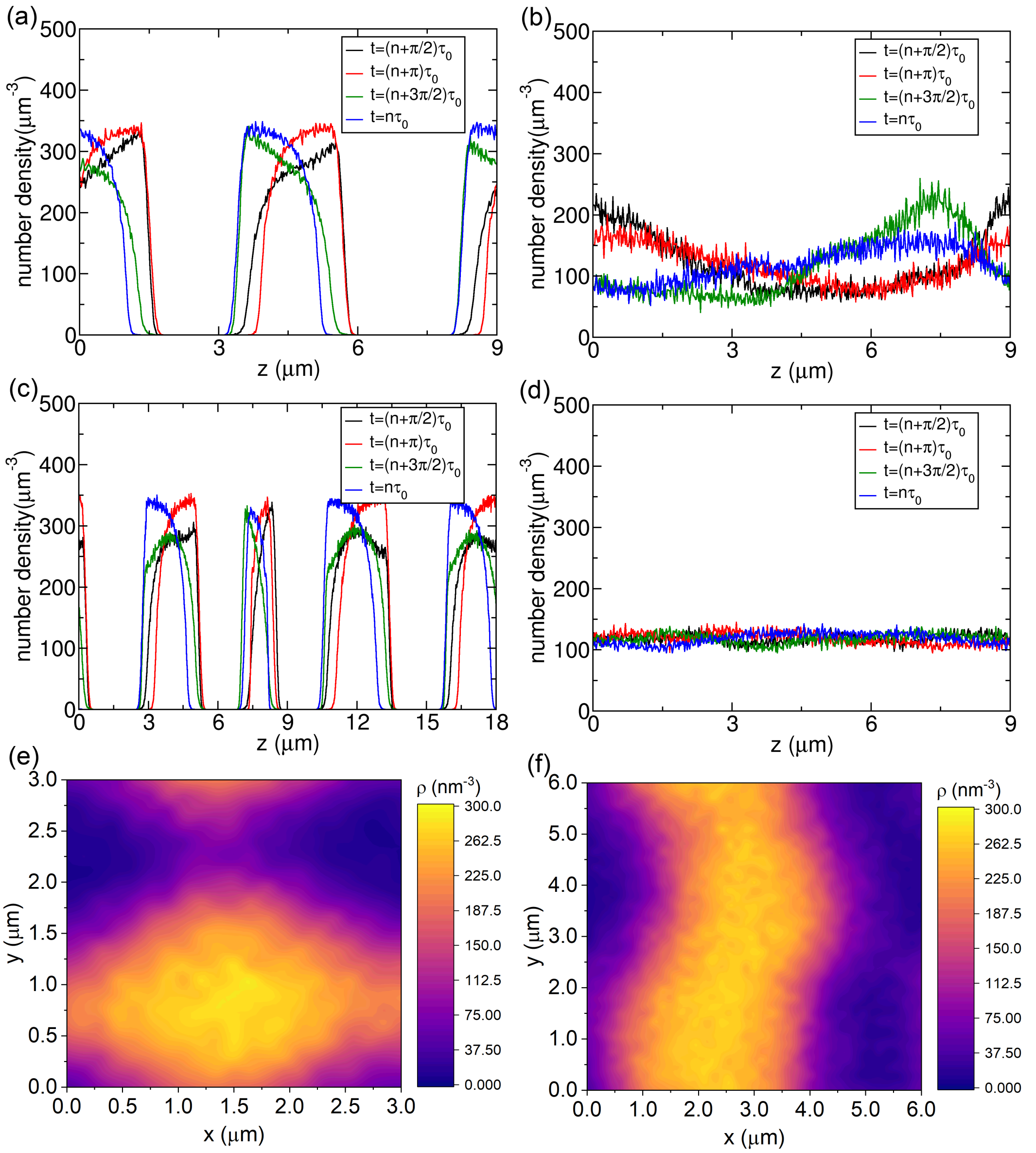

Figure S5: The density profiles of positively charged particles with 20e. (a) Result along field direction under 100 Hz at different moments. (b) Particles under 10 Hz at different moments. (c,d) Densities at different moments from enlarged systems corresponding to Figure S4a and b, respectively. (e) Density profile perpendicular to field (x-y) under 10 Hz. (f) Result from the broadening system of (e).

Spatial Pressure Profiles in Lane structure

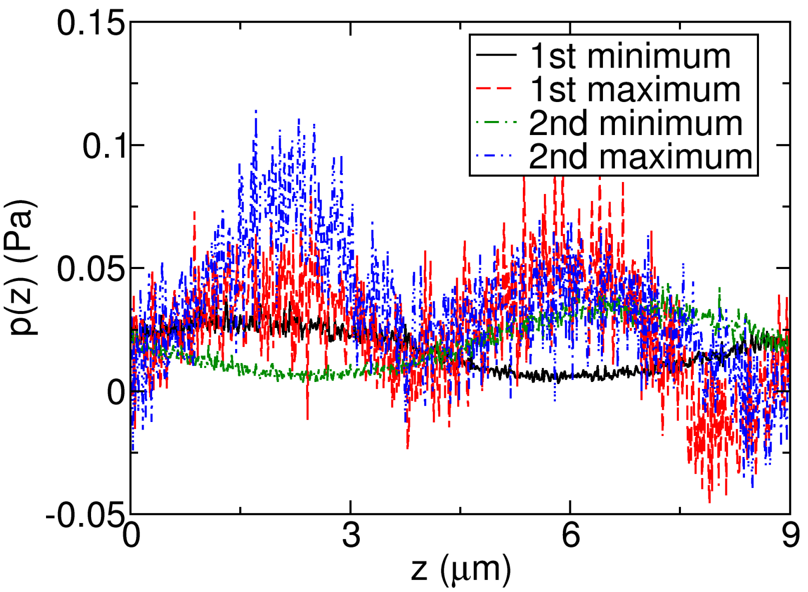

Figure S6: The pressure profiles $p(z)$ along AC field direction under $\omega = 10$ Hz at different times, which correspond to the two minima and maxima of p_z within an oscillating period.

Work and Heat Dissipation

The rates of work on the time steps dumped during simulations $\langle \dot{w}(t) \rangle = \langle \nabla U_i(t) \cdot \mathbf{F}_{ext,i}(t) \rangle / \gamma_i$ are shown in Figure S7, in order to provide more comprehensive view for the time evolutions of rates of work.

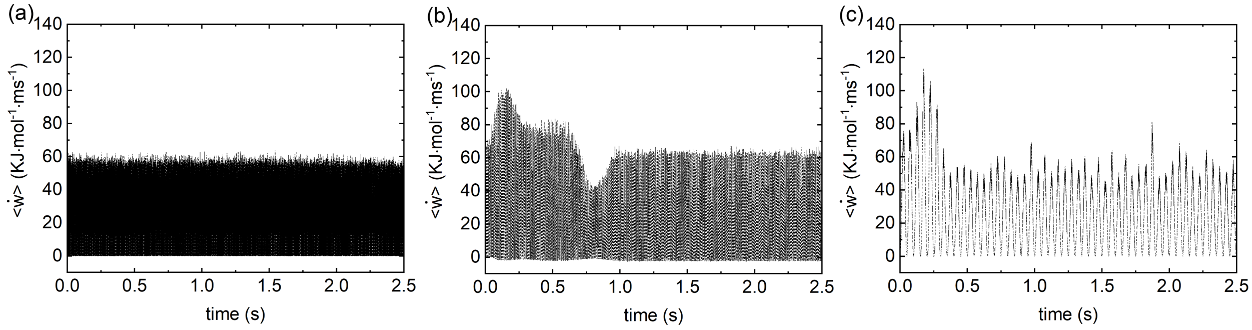

Figure S7: The rate of work $\langle \dot{w} \rangle$ *versus* simulation times for $\pm 20e$ charged particle systems. (a) Results under 1 kHz. (b) Results under 100 Hz. (c) Results under 10 Hz.

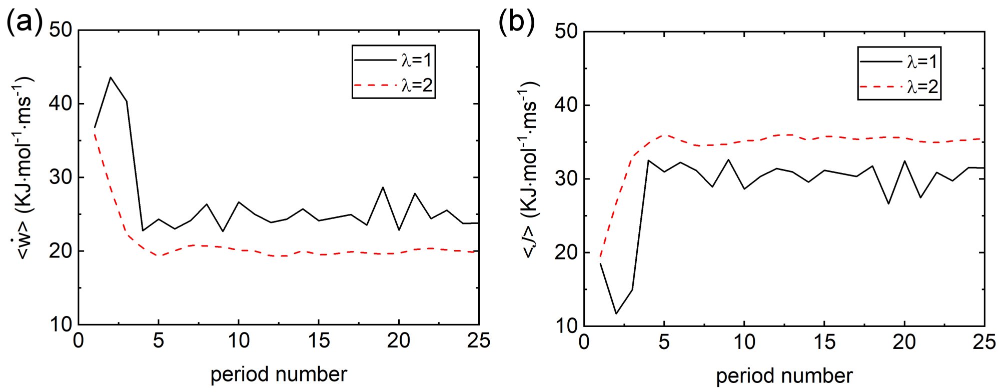

Figure S8: The rate of work $\langle \dot{w} \rangle$(a) and heat dissipation $\langle \mathcal{J} \rangle$(b) for the systems under 10 Hz. The dash lines are the results from the system with a box width scale by a factor $\lambda = 2$ ($L_x = L_y = 6\ \mu$m), the solid lines are from the original system with $L_x = L_y = 3\ \mu$m ($\lambda = 1$).

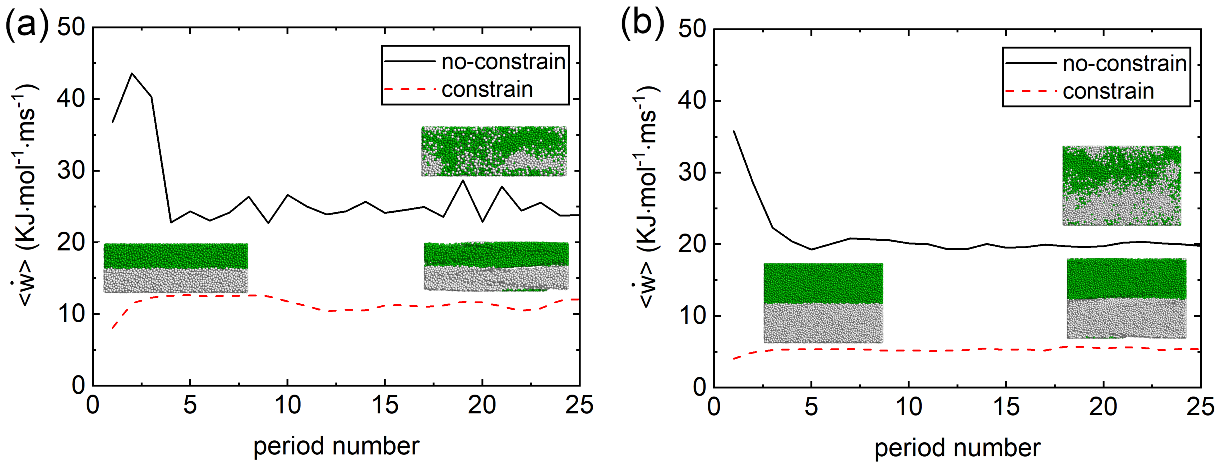

Figure S9: The rates of work in the system starting from laned structure and with constraints (red curves), and its comparison with the original system without constraint. (a) Results of system with smaller box width. (b) Results of system with larger box width by a factor of 2.

In order to investigate the influence of collision strength between interfaces on the rate of work $\langle \dot{w} \rangle$, we simulate a system with extremely weak collision. A phase-separated configuration like a lane-structure is constructed initially (the left snapshot in Figure S9a). Then we apply a harmonic restraint on x direction, so the colloids will fluctuate in a very small region on x direction, and the initial laned configuration will not be changed too much (snapshot in the lower-right corner of Figure S9a). The $\langle \dot{w} \rangle$ values are much lower than the original system without constraint, since the collision between the two lanes is much weaker.

The systems with larger box width (x and y) by a factor $\lambda = 2$ are also investigated with the similar approach as above. And the results are shown in Figure S9b. Similarly, the system with constraints shows smaller $\langle \dot{w} \rangle$ values, because of very weak collisions. All the two $\langle \dot{w} \rangle$ values are smaller than those shown in Figure S9a. So the $\langle \dot{w} \rangle$ for lane formation, is smaller as the system size is larger, as $\langle \dot{w} \rangle$ is a per particle quantity.
